# DFT-Based Elucidation and Evaluation of Selenium-Modified Tacrine Derivatives: Theoretical and Physicochemical Insights for Alzheimer’s Disease Therapy

**DOI:** 10.3390/molecules30122553

**Published:** 2025-06-11

**Authors:** Roberto Barbosa Morais, Manoela do Sacramento, Cecilia Scimmi, Darling de Andrade Lourenço, Frederico Schmitt Kremer, Lucielli Savegnago, Diego Alves

**Affiliations:** 1 LASOL-CCQFA, Universidade Federal de Pelotas—UFPel, P.O. Box 354-96010-900, Pelotas 96160–000, RS, Brazil; robertomorais500@gmail.com (R.B.M.); manoelasacramento@hotmail.com (M.d.S.); 2 Group of Catalysis, Synthesis and Organic Green Chemistry, Department of Pharmaceutical Sciences, University of Perugia, Via del Liceo 1, 06123 Perugia, Italy; cecilia.scimmi@dottorandi.unipg.it; 3 Laboratório de Bioinformática (Omixlab), Centro de Desenvolvimento Tecnológico (CDTec), Universidade Federal de Pelotas—UFPel, Pelotas 96160–000, RS, Brazil; darlinglourenco@gmail.com (D.d.A.L.); fred.s.kremer@gmail.com (F.S.K.); 4 Neurobiotechnology Research Group—GPN, Technological Development Center, Federal University of Pelotas—UFPel, Pelotas 96160–000, RS, Brazil

**Keywords:** selenium, acetylcholinesterase inhibitors, Alzheimer’s disease, molecular docking, density functional theory, pharmacokinetics, toxicology, neurodegenerative diseases, beta-secretases, computational drug design

## Abstract

The incorporation of selenium into tacrine derivatives has been explored as a novel strategy to enhance therapeutic efficacy while minimizing toxicity in the treatment of neurodegenerative diseases such as Alzheimer’s. This study utilized computational and experimental approaches, including Density Functional Theory (DFT), molecular docking, pharmacokinetic profiling, and toxicological predictions, to evaluate the potential of these derivatives. The selenium-modified compounds demonstrated improved electronic properties, such as narrower HOMO–LUMO gaps and optimized electronegativity, resulting in enhanced stability and reactivity. Pharmacokinetic analyses revealed favorable absorption, distribution, and blood–brain barrier penetration, while toxicological assessments indicated reduced hepatotoxicity and skin sensitization risks compared to tacrine. Molecular docking and dynamic simulations highlighted strong and stable interactions of the derivatives with critical enzymes, including acetylcholinesterase (AChE) and beta-secretases (BACE1 and BACE2). Compounds **12** and **13**, in particular, emerged as the most promising candidates due to their superior stability and binding affinity. These findings underscore the potential of selenium-modified tacrine derivatives as safer and more effective therapeutic agents for Alzheimer’s disease, warranting further experimental validation.

## 1. Introduction

Quantum mechanics (QM) has emerged as an indispensable tool in modern drug design, providing a rigorous framework for investigating the electronic structure and properties of molecules at the atomic and molecular levels [1]. By exploring quantum particles, scientists learn a lot about how molecules behave, change, and function in living things [2].

QM allows for the computation of many different molecular descriptors, such as electronic structures, bond orders, bond lengths, bond angles, or energy levels [3]. These descriptors are paramount when considering the performance of molecules in different environments, for example, in solution or within some biological systems [4]. Moreover, it is possible under quantum mechanics to compute such physicochemical properties as polarity, polarizability, ionization potential, electron affinity, solubility, and so on, which are very important for the absorption-distribution-metabolism excretion-toxicity (ADMET) profile of a drug [5].

In molecular modeling to study the relationship between the chemical structure and activity of compounds, electronic molecular descriptors obtained by Quantum Calculations and Density Functional Theory (DFT) calculations are used, especially B3LYP functionals with extended basis sets [6,7]. Reactivity indices are employed to compare antioxidant activity based on different reaction mechanisms. Bond Dissociation Energies (BDEs) are used to assess antioxidant activity via Hydrogen Atom Transfer (HAT) and help anticipate antioxidant activity via Simple Electron Transfer (SET) and other processes [8].

Other important parameters include Frontier Molecular Orbital Energies, such as Highest Occupied Molecular Orbital (HOMO) and the Lowest Unoccupied Molecular Orbital (LUMO), and gap energy, which indicate the intrinsic reactivity of the compounds [9]. The electron density of Mulliken charges is also considered to understand the stability and contributions of bioactive groups to antioxidant properties [10]. These approaches are crucial in understanding the mechanisms of biological activity and action of antioxidants and in the search for compounds with relevant pharmaceutical activities [11].

Tacrine, a potent and reversible acetylcholinesterase inhibitor, was the pioneering drug approved for Alzheimer’s disease (AD) treatment. While clinical trials demonstrated efficacy, its therapeutic use was discontinued due to severe hepatotoxicity. Rapidly metabolized by cytochrome P450 (CYP) complex enzymes [12], tacrine’s primary metabolic pathway involves hydroxylation by CYP1A2, resulting in the formation of 1-, 2-, 4-, and 7-hydroxy metabolites. Accumulating evidence strongly implicates the 7-hydroxytacrine metabolite as the primary culprit in tacrine-induced hepatotoxicity [13]. This metabolite is believed to serve as a precursor to a highly reactive quinone derivative, which is thought to be the ultimate causative agent of liver damage [14].

Selenium-containing compounds have emerged as a focal point within medicinal chemistry research [15]. These molecules have garnered significant attention due to their pharmacological potential [16]. Notably, several studies have demonstrated that selenium compounds exhibit catalytic properties analogous to glutathione peroxidase, an enzyme pivotal in the cellular defense against oxidative stress through the reduction of hydroperoxides and peroxynitrites [17]. Furthermore, selenium is recognized as an essential micronutrient for optimal brain function and has shown promise in mitigating certain neurological pathologies prevalent in the elderly population [18]. Recently, our research group demonstrated the synthesis and conducted preliminary in silico and in vitro studies on the toxicology and antioxidant properties of selenylated analogues of tacrine [19]. However, further research is necessary to fully comprehend the safety and efficacy of these Se-THA hybrids, as well as to determine safe and effective therapeutic doses.

**Figure 1:**
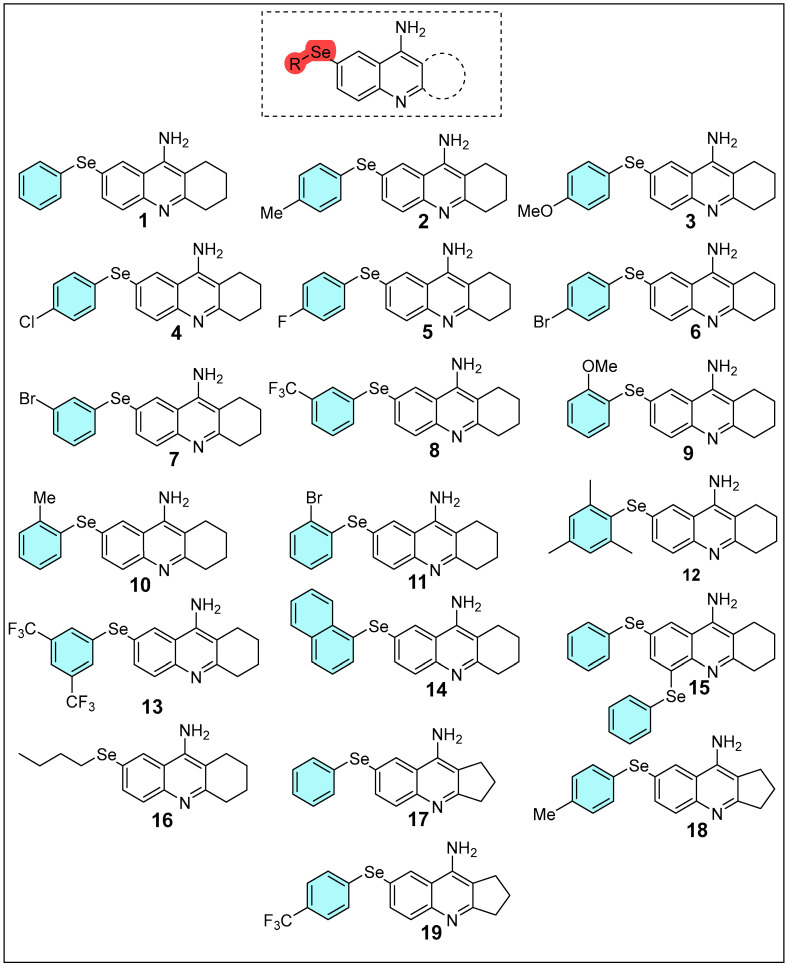
Structure of tacrine derivatives examined in this study.

Hence, this study employs computational approaches, including DFT, molecular docking, and molecular dynamics simulations, combined with pharmacokinetic and toxicological analyses, to investigate the electronic properties, stability, reactivity, and molecular interactions of selenium-modified tacrine derivatives Figure 1. The goal is to design and evaluate derivatives with enhanced therapeutic efficacy and reduced toxicity for neurodegenerative diseases like Alzheimer’s. This strategy involves the rational incorporation of electron-donating and electron-withdrawing substituents, as well as selenium, into the tacrine scaffold to optimize physicochemical properties and interactions with the biological target.

## 2. Results and Discussion

### 2.1. DFT Studies

The pursuit of improving the therapeutic effectiveness and reducing the toxicity of tacrine derivatives led to a comprehensive investigation focusing on compounds containing an organic selenium component [20]. This study sought to address the limitations of traditional tacrine formulations, particularly their hepatotoxicity, by leveraging selenium’s unique properties, which include antioxidative and catalytic potential akin to glutathione peroxidase [19].

The research commenced with extensive theoretical studies to explore the mechanisms of action of these selenium-containing compounds. By employing advanced computational methods, including DFT, the study investigated molecular descriptor energies such as HOMO, LUMO, and their associated energy gaps [21] These descriptors provided critical insights into the compounds*’* stability, reactivity, and potential for interaction with biological targets. The DFT analyses also allowed the prediction of electron donation and reception capabilities, shedding light on how these compounds might interact with active sites in acetylcholinesterase, the key enzyme targeted in Alzheimer’s disease therapy [22].

A pivotal aspect of the study was the optimization of synthetic routes for producing these novel tacrine derivatives. This optimization involved identifying favorable reaction conditions, selecting appropriate precursors, and integrating selenium atoms into the molecular framework. The process was informed by Quantitative Structure-Activity Relationship (QSAR) analyses, molecular docking studies, and pharmacokinetic simulations. QSAR provided a quantitative link between chemical structures and their biological activities, enabling the prioritization of compounds with the highest potential efficacy [23].

Molecular docking studies revealed how these derivatives might interact with acetylcholinesterase at the molecular level, highlighting binding affinities and the specific interactions of selenium atoms within the active site. Toxicological predictions were performed to evaluate the potential risks associated with these compounds, including hepatotoxicity and mutagenicity, ensuring that the synthesized derivatives maintained a favorable safety profile [24].

To validate the theoretical findings, some experimental studies were carried out in parallel, such as toxicity and antioxidant tests for compound **1**; the results were previously published by our group [19]. Initial efforts focused on synthesizing compounds that exhibited the most promising profiles in silico. The synthesized derivatives will be subjected to rigorous in vitro testing to evaluate their ability to inhibit acetylcholinesterase and reduce oxidative stress, which is a hallmark of Alzheimer’s pathology [25]. Encouraging candidates from in vitro tests advanced to in vivo studies to assess their pharmacokinetics, bioavailability, and overall therapeutic efficacy. The integration of selenium into the tacrine scaffold proved to be particularly beneficial, as selenium’s antioxidative properties appeared to mitigate oxidative damage, a significant contributor to neuronal degeneration [26].

Another critical dimension of the research was the analysis of electronic properties, specifically focusing on the distribution of the HOMO and the LUMO. These properties are crucial for understanding the compounds’ reactivity and stability. The study revealed that the presence of selenium significantly influenced the electronic distribution, directing the HOMO electron density toward the aromatic ring containing selenium. This redistribution is believed to enhance the compound’s ability to interact with biological targets while improving stability. Interestingly, substituents on the tacrine scaffold had a relatively lesser impact on electronic distribution compared to selenium, underscoring the unique role of this element in modulating molecular properties [27].

Moreover, pharmacokinetic profiling of the derivatives provided insights into their absorption, distribution, metabolism, and excretion (ADME) properties. The use of computational tools like PreADMET allowed for predictions regarding human intestinal absorption, blood–brain barrier penetration, and interactions with cytochrome P450 enzymes. These analyses confirmed that the incorporation of selenium not only improved the compounds’ bioavailability but also reduced the possibility of forming toxic metabolites, addressing one of the key challenges associated with tacrine use [28].

The preliminary results reported by us [19] form part of this broader study, which provides a detailed comparison of the electronic properties of selenium-modified tacrine derivatives with different substituents and unmodified tacrine.

**Figure 2:**
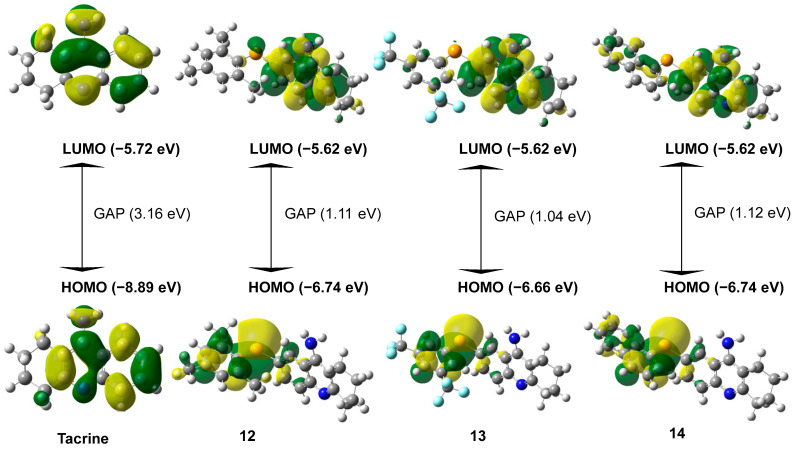
Influence of Substituents and Selenium on HOMO–LUMO Electronic Distribution and GAP the difference between LUMO and HOMO (LUMO-HOMO) in Tacrine Derivatives.

Figure 2 presents the results for the most promising compounds. The tacrine derivatives (compounds **12**–**14**) exhibit HOMO energy levels ranging from −6.66 eV to −6.74 eV, which are significantly higher than that of the original tacrine molecule (−8.89 eV). This difference indicates a lower ionization potential, suggesting that these derivatives can more readily donate electrons [29]. As a result, they may interact more effectively with biological targets through electron donation. The similar HOMO levels observed among the selenium-containing compounds highlight the influence of selenium on these electronic properties [30].

The LUMO energy levels remain relatively consistent across the derivatives, averaging around −5.62 eV, which is slightly higher than that of the parent tacrine molecule (−5.72 eV). This consistency suggests that the electron-accepting capability is largely preserved. Notably, selenium appears to influence the spatial distribution of LUMO density, shifting it toward regions potentially involved in interactions with acetylcholinesterase, which may enhance binding affinity [27].

A key observation from Figure 2 is that the HOMO–LUMO energy gap in the tacrine derivatives is significantly reduced, averaging around 1.1 eV, compared to 3.16 eV in the parent tacrine molecule. This narrower energy gap suggests increased chemical reactivity and enhanced charge-transfer capability, which are relevant features for potential enzyme inhibition mechanisms [28].

Although not shown in the figure, the electronegativity values of the derivatives were previously reported by Sacramento et al. (2024) [19]. These values are lower (5.93–6.18 eV) than that of the parent tacrine molecule (7.30 eV), further supporting their potential for improved interactions in biological systems. Selenium plays a key role in this decrease, contributing to the development of compounds that may act on multiple biological pathways. The incorporation of selenium into the tacrine scaffold significantly alters the electronic distribution of the derivatives [31].

**Figure 3:**
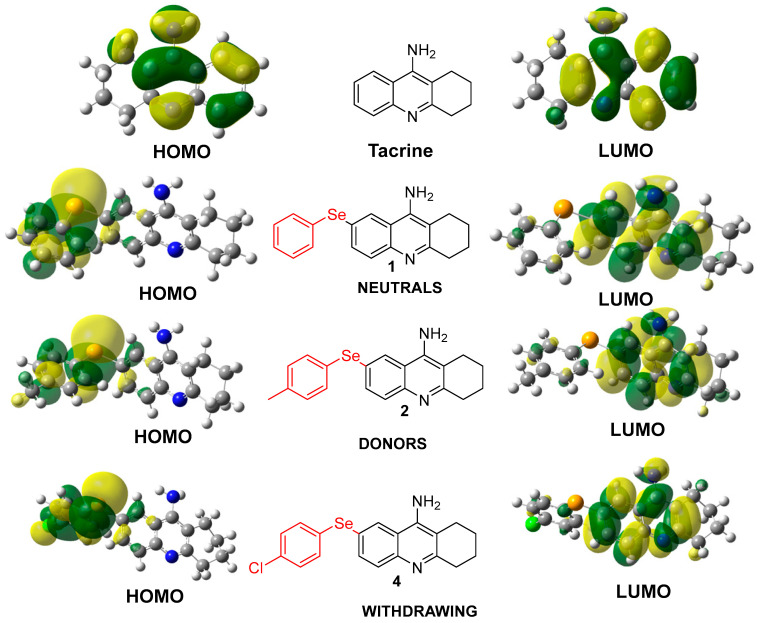
Electronic Distribution of HOMO and LUMO in Tacrine and its Selenium-Modified Derivatives.

The incorporation of selenium into tacrine derivatives significantly alters the electron distribution, particularly within the HOMO orbitals. As shown in Figure 3, selenium-containing compounds (**1**, **2**, and **4**) exhibit a pronounced localization of HOMO electron density on the aromatic ring bearing the selenium atom, in contrast to the parent tacrine molecule, where the HOMO is more evenly distributed. This shift enhances the electron-donating capability of the selenium moiety, potentially improving interactions with biological targets such as acetylcholinesterase. While substituents like methyl groups (electron-donating) or halogens (electron-withdrawing) exert some influence, their effects are modest when compared to the substantial impact of selenium. Overall, selenium plays a pivotal role in modulating the reactivity and interaction potential of these derivatives [32].

The comparison with tacrine underscores the significant improvements achieved through molecular modification. The original tacrine, with its high GAP and electronegativity, exhibits lower reactivity and limited interaction potential with biological targets. In contrast, the derivatives demonstrate optimized electronic properties, making them more suitable for therapeutic applications. The reduced GAP and electronegativity in the derivatives align with the goal of enhancing efficacy while minimizing toxicity [33].

The incorporation of selenium and the structural modifications to the tacrine derivatives have significantly influenced their electronic properties. The data indicate that these changes lead to improved HOMO–LUMO energy levels and a reduced energy gap, suggesting increased molecular reactivity. This enhances their potential to interact with biological targets, as supported by molecular orbital analysis and other reactivity parameters [9]. These findings provide a strong foundation for further experimental validation and highlight the potential of selenium-containing tacrine derivatives as promising candidates for Alzheimer’s disease therapy. By systematically analyzing these electronic parameters, the study offers valuable insights into the rational design of safer and more effective drugs.

The electronic properties of the tacrine derivatives and unmodified tacrine, as depicted in Figure 3, reveal critical insights into their stability, reactivity, and potential therapeutic application. A detailed analysis of these compounds’ HOMO and LUMO energies provides a deeper understanding of their electronic distributions, which significantly influence their behavior in biological systems [27].

The GAP between HOMO and LUMO is a crucial parameter for determining a compound’s chemical reactivity. In this study, the derivatives exhibit relatively lower energy gaps, suggesting increased chemical reactivity compared to tacrine [10].

The energy gap also influences the compounds’ electronic excitation and charge transfer potential. For instance, selenium-containing compounds appear to direct the HOMO and LUMO electron densities toward specific regions of the molecule, optimizing their interactions with the biological target [34]. This targeted electronic distribution enhances the therapeutic potential of these derivatives while minimizing off-target effects [12].

The electronegativity values of the tacrine derivatives are relatively consistent, indicating comparable tendencies to attract electrons. This uniformity suggests that the introduction of selenium and substituents does not drastically alter the electron-attracting capabilities of the molecules, preserving their chemical behavior [35].

Interestingly, tacrine exhibits a significantly higher electronegativity value compared to the derivatives. This observation suggests that the derivatives are more stable than tacrine, as lower electronegativity values generally indicate a reduced likelihood of engaging in rapid, potentially harmful electron transfer reactions. The improved stability of the derivatives supports their potential as safer alternatives to tacrine, especially in mitigating the hepatotoxic effects associated with tacrine’s metabolism [36].

Figure 3 highlights how selenium and various substituents influence the electronic distribution within the tacrine scaffold. Selenium, known for its unique electron-donating and antioxidative properties, significantly impacts the HOMO electron density, directing it toward the aromatic ring containing selenium. This redistribution enhances the molecule’s ability to engage in specific interactions with biological targets, such as acetylcholinesterase, while maintaining overall molecular stability. Substituents, categorized as electron-donating or electron-withdrawing groups, further modulate the electronic properties [27].

- **Neutral substituents (Compound 1):** Exhibit balanced HOMO–LUMO distributions, indicating moderate stability and reactivity.
- **Electron-donating substituents (Compound 2):** Slightly reduce the energy gap, enhancing reactivity while maintaining sufficient stability for biological applications.
- **Electron-withdrawing substituents (Compound 4):** Increase electronegativity and stabilize the LUMO, potentially reducing off-target interactions.

These variations in electronic behavior highlight the flexibility of the tacrine scaffold in accommodating diverse chemical modifications to optimize therapeutic properties [37].

The comparison between tacrine and its selenium-containing derivatives reveals clear improvements in electronic properties resulting from structural modifications. Tacrine exhibits the highest electronegativity (7.30 eV) and the widest HOMO–LUMO gap (3.16 eV), suggesting lower chemical reactivity and a potential for toxicity. Notably, this may be linked to a specific point of electronic instability at carbon 7, which is effectively stabilized in the proposed derivatives through the incorporation of selenium at that position [29].

On the other hand, the derivatives—particularly those containing selenium—exhibit significantly lower HOMO–LUMO gap values (0.81–1.15 eV) and reduced electronegativity (5.93–6.18 eV). These features suggest increased chemical reactivity and an enhanced ability to interact with biological targets. Such electronic modifications highlight the potential of these molecules as more effective candidates for the treatment of Alzheimer’s disease [38].

Specifically, tacrine derivatives exhibited a distinctive distribution pattern, with HOMO electron density situated between the selenium atom and the substituent ring. Meanwhile, the LUMO was predominantly located on the amino-acridine ring. In summary, while aniline derivatives tended to draw energy from the HOMO orbital, selenium-containing compounds directed HOMO density towards the selenium atom and the adjacent ring. These findings provide valuable insights into the design and development of novel therapeutic agents with improved efficacy and safety profiles [10].

In order to verify whether it was really the influence of selenium, we analyzed a derivative replacing selenium with the oxygen atom, which has similar characteristics. We observed that it contains different HOMO profiles than derivatives containing selenium (Figure S1; Supplementary Materials).

**Figure 4:**
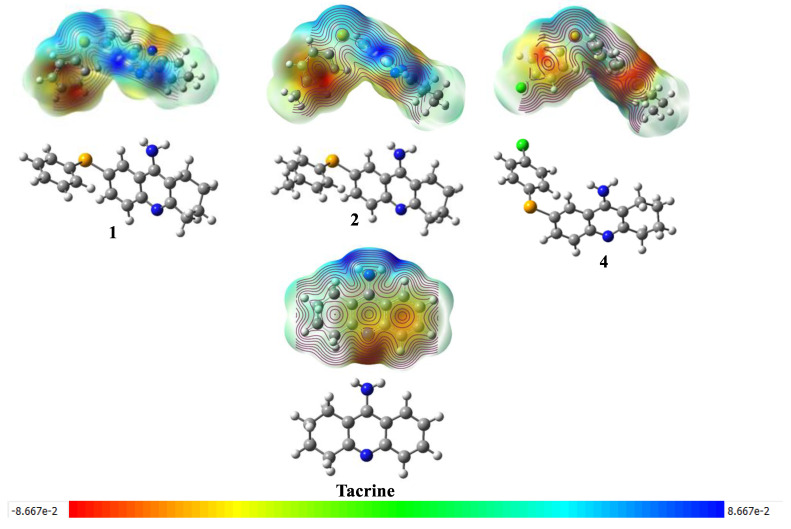
Molecular Electrostatic Potential (MEP) Maps of Tacrine and Its Selenium-Modified Derivatives.

MEPs offer a valuable visual representation of electron density distribution within a molecule. Regions of red and blue in these maps correspond to areas of high (negative) and low (positive) electron density, respectively (Figure 4). By examining MEPs of compounds in their ground states, we can effectively compare the influence of various substituents on molecular charge distribution [18].

Compound **1** exhibits negative regions near the hydrogen atoms, suggesting a slightly acidic character at these sites. Conversely, the most positive regions are likely associated with heteroatoms or the π system of the aromatic ring, implying basic or nucleophilic properties. Compound **2** demonstrates a more polarized charge distribution, with distinct regions of positive and negative charge. The selenium atom, for instance, is likely associated with a region of partial positive charge [39]. Similarly, compound **4** presents a polarized charge distribution, with the halogen atom bearing a partial negative charge. This observation highlights that, unlike the HOMO and LUMO maps, substituents significantly influence the distribution of electrostatic potential.

### 2.2. Pharmacokinetic Properties

The data presented in Table 1 provide a comprehensive overview of the pharmacokinetic properties of tacrine and its selenium-modified derivatives, highlighting key parameters such as HIA (%), BBBP (Log*CC*), permeability in the central nervous system (CNS) (Log*PS*), permeability across membranes (Log *P* 8 × 10^−6^ ca^−2^ nm/s), and the Log*P* values. These parameters are critical in understanding the absorption, distribution, metabolism, and excretion (ADME) characteristics of the compounds, ultimately influencing their therapeutic potential [16].

The HIA (%), which measures the intestinal absorption, demonstrates that most of the selenium-modified derivatives exhibit improved absorption compared to tacrine, which has an HIA of 94.17%. Notably, compounds such as 3, 9, and 15 achieve near-perfect absorption, with values of 99.65%, 99.94%, and 100%, respectively. This enhanced absorption indicates the potential for these derivatives to exhibit better bioavailability than tacrine, making them promising candidates for oral administration [18].

The BBBP (Log*CC*) values, which reflect the plasma protein binding capacity, vary across the derivatives. Tacrine exhibits a relatively high BBBP value of 0.21, suggesting significant plasma protein interaction, which can influence the drug’s free concentration in circulation. Several derivatives, such as compounds **3** and **15**, display negative BBBP values, indicating reduced protein binding. This characteristic could enhance the free drug fraction, potentially improving therapeutic efficacy. However, derivatives with higher PBH values, such as compound **17** (0.12), may exhibit prolonged circulation times due to stronger protein binding [36].

The CNS permeability (Log*PS*) values offer insights into the compounds’ ability to cross the blood–brain barrier (BBB), a crucial factor for neuroactive drugs targeting central nervous system disorders like Alzheimer’s disease. Tacrine shows a Log*PS* of −1.70, indicating limited BBBP penetration. In comparison, most derivatives exhibit similar or slightly better permeability values, with compound **15** demonstrating the least restriction at −1.13. This improvement suggests that the selenium-modified derivatives may have enhanced potential for effective central nervous system delivery [40].

The membrane permeability (Log*P* 8 × 10^−6^ ca^−2^ nm/s) values further emphasize the enhanced transport properties of the derivatives. Tacrine demonstrates a permeability value of 1.58, while the derivatives show comparable or slightly lower values, indicating similar abilities to permeate cellular membranes. Compound **1**, with a permeability of 1.54, closely matches tacrine’s performance, whereas compound **12** exhibits a lower value of 0.98, which might suggest reduced membrane-crossing efficiency for this specific derivative [23].

The Log *P* values, a measure of lipophilicity, are critical for understanding the compounds’ solubility and membrane permeability. Tacrine has a Log*P* of 2.83, indicating moderate lipophilicity. In contrast, most derivatives exhibit higher Log *P* values, ranging from 3.51 to 5.75. Compounds like **13** (5.75) and **12** (5.39) are particularly lipophilic, which could enhance their ability to penetrate lipid-rich environments such as the BBB. However, excessively high lipophilicity might also pose challenges related to reduced solubility in aqueous environments and potential toxicity [34].

Overall, the pharmacokinetic analysis underscores the improvements achieved in the selenium-modified derivatives compared to tacrine. Enhanced AIH values indicate better absorption, while variations in PBH and permeability values suggest that the derivatives have been optimized for better bioavailability and central nervous system targeting. The increased Log *P* values highlight the improved lipophilicity of the derivatives, which could contribute to their therapeutic efficacy. These findings provide valuable insights for selecting the most promising candidates for further in vitro and in vivo evaluations, paving the way for the development of safer and more effective treatments for neurodegenerative diseases like Alzheimer’s [35].

### 2.3. Toxicological Properties

Table 2 presents toxicological predictions for tacrine and its selenium-modified derivatives, focusing on three critical parameters: carcinogenicity in humans, hepatotoxicity in humans, and skin sensitization. These parameters are pivotal in assessing the safety profiles of potential drug candidates and ensuring that modifications do not introduce unacceptable risks [36].

For carcinogenicity, the majority of the derivatives are classified as “plausible” for their potential to induce carcinogenic effects in humans. This classification suggests that while the risk exists, it is not definitive and warrants further investigation. Compounds **8**, **13**, **14**, and **15** stand out as exceptions, with no evidence or indication of carcinogenic potential. This distinction highlights these derivatives as potentially safer options in terms of long-term use, particularly when compared to tacrine, which is also categorized as “plausible” for carcinogenicity. The widespread plausible classification across the derivatives emphasizes the need for additional preclinical and clinical studies to confirm the safety of these compounds [41].

For hepatotoxicity, tacrine is distinctly marked as “certain”, reflecting its well-documented hepatotoxic effects, which have been a significant limitation in its therapeutic use. In contrast, none of the selenium-modified derivatives exhibit indications of hepatotoxicity. This stark difference underscores the success of the structural modifications, particularly the incorporation of selenium, in mitigating the hepatotoxic effects traditionally associated with tacrine. This improvement aligns with the broader goal of developing safer acetylcholinesterase inhibitors for Alzheimer’s disease [23].

Regarding skin sensitization, most compounds show no signs of inducing skin sensitization, with only compounds **3** and **9** marked as “plausible”. Tacrine, on the other hand, is categorized as “probable”, indicating a higher probability of causing adverse skin reactions compared to the derivatives. The limited instances of plausible skin sensitization among the derivatives suggest that selenium incorporation does not significantly increase the risk of allergic reactions through skin contact, further supporting their safety profiles [34].

Overall, the toxicological predictions indicate that the selenium-modified derivatives offer significant improvements over tacrine in terms of reduced hepatotoxicity and skin sensitization risks. However, the potential carcinogenicity of several derivatives remains a concern that must be addressed through further testing. Compounds such as **8**, **13**, **14**, and **15**, which show no indication of carcinogenicity, hepatotoxicity, or skin sensitization, emerge as particularly promising candidates for further development. These findings highlight the potential of selenium-modified tacrine derivatives to serve as safer alternatives for the treatment of neurodegenerative diseases, paving the way for further experimental validation and optimization [35].

### 2.4. Metabolic Prediction

Employing the ADMET Predictor^®^, a metabolic prediction for tacrine was conducted. The prediction identified potential metabolic pathways and primary metabolites (M1a, M2a, M3a, and M4a) derived from the parent molecule. Tacrine was predicted to be extensively metabolized by CYP1A2 and CYP2D6 enzymes [34].

**Figure 5:**
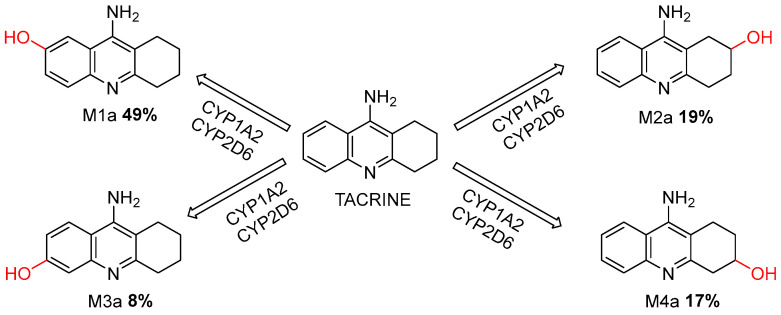
Metabolic Pathways of Tacrine Mediated by CYP1A2 and CYP2D6 Enzymes.

These enzymes catalyze structural modifications, resulting in the formation of metabolites M1a, M2a, M3a, and M4a. As aligned with previous literature, M1a, the predominant metabolite, is recognized as the primary reactive intermediate responsible for toxicity. Subsequent metabolites include M2a, M3a, and M4a (Figure 5).

We performed a metabolic prediction for our compounds that showed better theoretical results. Metabolic prediction studies revealed that our compounds exhibited favorable theoretical outcomes in comparison to tacrine. Notably, the likelihood of forming reactive intermediates was significantly reduced. The primary metabolic transformation observed was the substitution of one methyl group with a hydroxyl group (-OH) [30].

**Figure 6:**
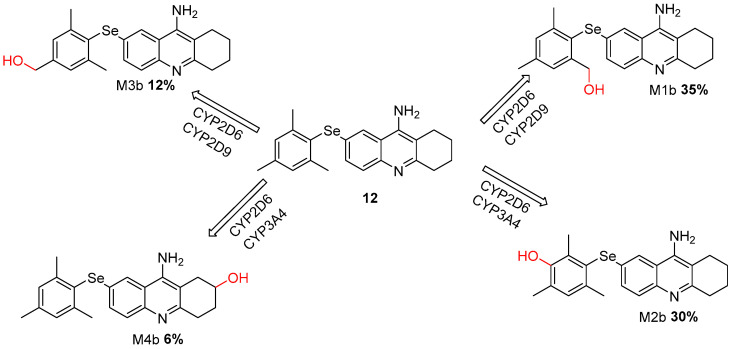
Metabolic Pathways of Selenium-Modified Tacrine Derivative (Compound **12**) Mediated by CYP2D6, CYP2D9, and CYP3A4 Enzymes.

We conducted a comprehensive analysis of the metabolic profiles of all proposed derivatives and compared them with the metabolic profile of tacrine in the context of the CYP enzyme complex. Our evaluation revealed a significant formation of the probable metabolites M1b and M2b in compound **12** (Figure 6), accounting for 35% and 30% of the total metabolic products, respectively [40].

**Figure 7:**
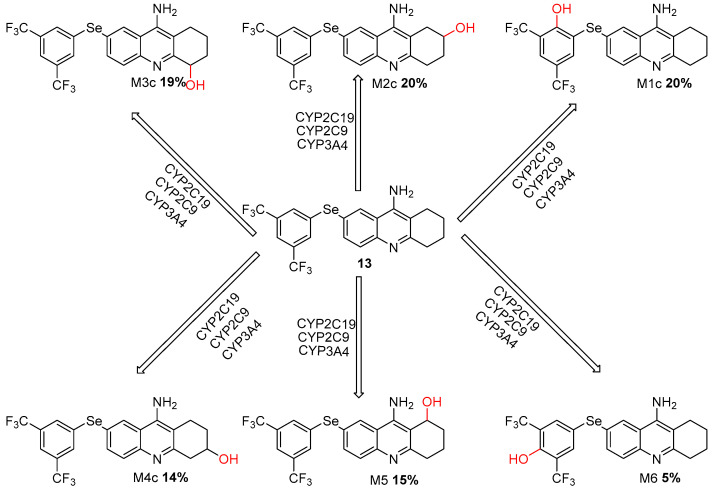
Metabolic Pathways of Selenium-Modified Tacrine Derivative (Compound **13**) Mediated by CYP2C19, CYP2C9, and CYP3A4 Enzymes.

Primarily catalyzed by CYP2C19, CYP2C9, and CYP3A4 isoforms, the biotransformation of compound **13** resulted in multiple oxygenated metabolites, of which M1c and M2c, each with 20%, emerged as the major metabolites Figure 7.

This finding suggests that the metabolic pathways catalyzed by CYP2D6, CYP2D9, and CYP3A4 are predominantly utilized during the biotransformation of these compounds. Notably, despite the high metabolic activity observed, the metabolites generated from these derivatives do not exhibit structural characteristics typically associated with hepatotoxicity. This observation highlights the potential safety of these compounds, as the absence of hepatotoxic metabolites could reduce the possibility of adverse liver-related effects, a common concern in the development of compounds targeting the CYP complex [23].

### 2.5. Molecular Docking

The interaction energy of the receptor–ligand complex was estimated for all planned compounds. The compounds were subjected to molecular docking for AChE and its isoform Butyrylcholinesterase (BuAChE), as well as for BACE1 and its isoform β-secretase 2 (BACE2). Thus, the results are demonstrated according to the score values shown in Table 3. The entire docking methodology was validated using redocking with known and co-crystalized inhibitor compounds (Figure S2; Supplementary Materials).

By analyzing the data presented in Table 3, a distinct interaction pattern was observed for compounds **11** to **15**. These compounds demonstrated superior binding energy scores compared to others, indicating a stronger and more favorable interaction. This trend was consistent across both the primary targets and their respective isoforms, suggesting a high degree of specificity and affinity in their binding behavior. Such results highlight the potential of these compounds for further development as selective inhibitors or modulators [10].

**Figure 8:**
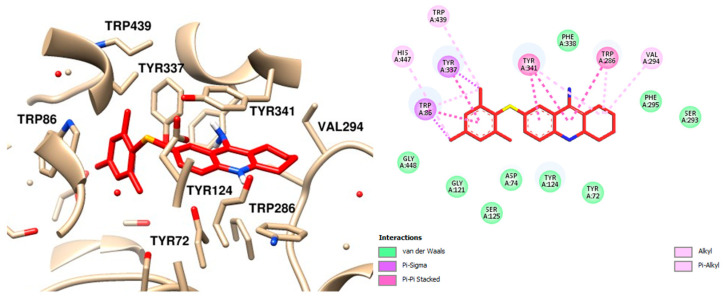
Molecular Docking Analysis Interaction Map of Compound **12** and AChE, the Best Receptor Ligand Orientation and 2D Interaction, Highlighting the Main Interactions in the Active Site.

Upon analyzing the interaction of the compounds, it was observed that compound **12**, which contains a mesityl group, exhibited the lowest interaction energy. Notably, the mesityl group interacts with the amino acids substituted with an aromatic ring at the anionic site. These interactions include π-sigma interactions with Trp86 and Tyr337, as well as a π-alkyl interaction with the catalytic amino acid His447. Additional hydrophobic interactions with other amino acids within the active site were also identified (Figure 8) [38].

**Figure 9:**
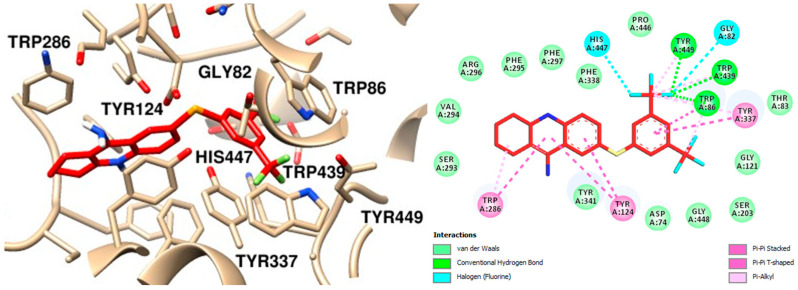
Molecular Docking Analysis: Binding Interactions of Tacrine Derivative **13** with AChE Active Site Residues.

Another compound that exhibited significant interaction was compound **13**, which also demonstrated one of the lowest interaction energy scores. Notably, this compound formed conventional hydrogen bonds with key amino acids within the active site, including Trp86, Trp439, and Tyr449, which are critical for the enzyme’s catalytic function. In addition to these central interactions, compound **13** also engaged in several other interactions with amino acids located in peripheral regions of the binding site. These peripheral interactions, although less prominent, may contribute to the overall stability and specificity of the binding (Figure 9) [40].

### 2.6. Molecular Dynamics

To ensure the reliability of our results and validate the system’s stability, we combined molecular docking with molecular dynamics (MD) simulations. The docking results for the complexes AChE–tacrine, AChE–compound **12**, and AChE–compound **13**, as well as the AChE apoprotein, were subjected to 100 ns MD simulations to analyze their atomic behavior in detail. Root-mean-square deviation (RMSD), root-mean-square fluctuation (RMSF), and the number of hydrogen bonds were calculated for the three complexes. Simulations of the AChE apoprotein were also conducted to better understand its intrinsic atomic behavior. The RMSD results from triplicate simulations of the protein backbone for each system revealed that the AChE apoprotein tends to stabilize around 40 ns, with RMSD values ranging between 0.4 and 0.6 nm. This indicates overall structural stability in the absence of ligands (Figure S3A, Supplementary Materials). A similar stabilization pattern was observed in the RMSD for the protein backbones for systems with ligands (Figure S3B,D,F; Supplementary Materials).

Analysis of the RMSD for ligand heavy atoms fitted to the protein backbone showed distinct behaviors among the three ligands (Figure S3C,E,G; Supplementary Materials). The results were consistent among triplicates for compound **12**, while slightly more pronounced fluctuations were observed for tacrine and compound **13**. These findings suggest that all three ligands exhibit some degree of movement within the binding site, with compound **13** demonstrating the highest stability. Overall, the RMSD data highlight the stability and conformational dynamics of AChE when bound to different ligands. While each ligand induces some level of fluctuation, the protein structure remains stable, indicating effective binding without major disruptions.

**Figure 10:**
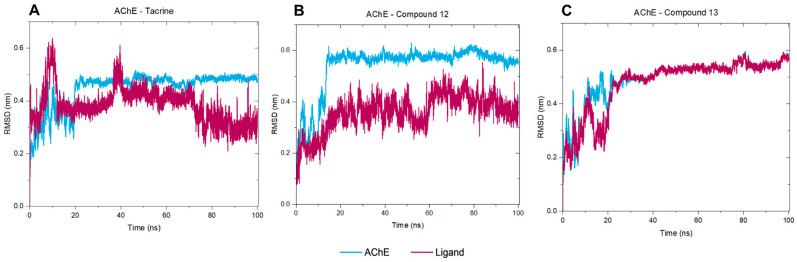
RMSD Analysis of AChE Complexes with Tacrine (**A**), Compound **12** (**B**), and Compound **13** (**C**). The RMSD of the AChE Protein Backbone (Fitted to Its Own Backbone) Is Shown in Blue, While the RMSD of Ligand Heavy Atoms (Fitted to the Protein Backbone) is Shown in Magenta.

In summary, considering the AChE–tacrine complex, the protein backbone stabilizes around 0.4 nm after 20 ns, and the heavy atoms from the ligand stabilize at 0.4 nm after 40 ns, indicating initial instability followed by stability (Figure 10A). Regarding the selenylated compound **12**, both AChE and the ligand quickly stabilize around 0.4 nm, indicating a stable complex throughout the simulation (Figure 10B), and a similar behavior was observed in the AChE–compound **13** complex (Figure 10C). Comprehensively, the RMSD data indicate that all three ligands show initial fluctuations, which stabilize over time. Nonetheless, both compounds **12** and **13** show similar stability profiles, suggesting that they form more stable complexes with AChE compared to tacrine. The stabilization of RMSD values for the ligands implies that they remained within the binding site throughout the simulation period, without significant dissociation.

**Figure 11:**
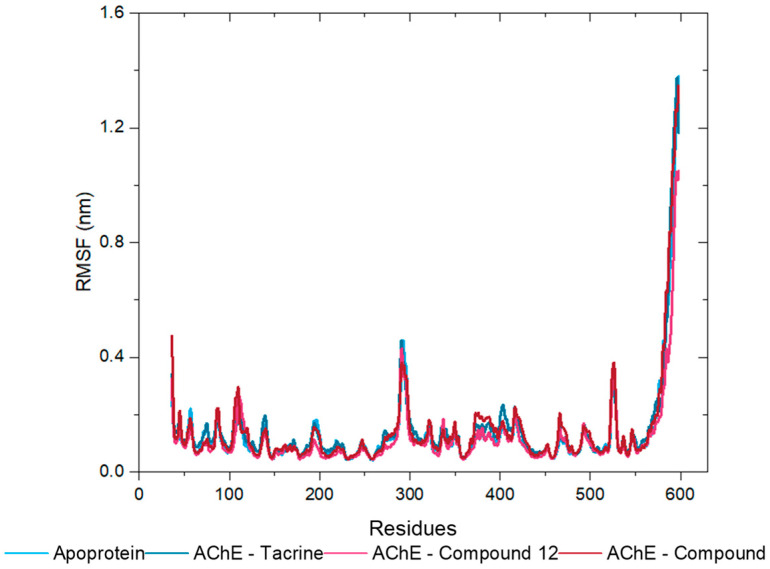
RMSF Analysis of Protein Backbone of AChE Apoprotein (Soft Blue Line) and Its Complexes with Tacrine (Dark Blue Line), Compound **12** (Pink Line), and Compound **13** (Red Line).

The root-mean-square fluctuation (RMSF) values were calculated to assess the flexibility of the protein chain residues during the MD simulations. RMSF quantifies the average deviation of each atom’s position from its mean position over the course of a simulation or within an ensemble of structures [31]. The RMSF values obtained across all triplicate runs for the AChE apoprotein (Figure S4A, Supplementary Materials) and its complexes with tacrine (Figure S4B, Supplementary Materials), compound **12** (Figure S4C, Supplementary Materials), and compound **13** (Figure S4D, Supplementary Materials) indicate that most residues exhibit low fluctuation, reflecting structural stability. However, increased flexibility was observed in the region around residue 600 across all systems, consistent with the dynamic nature of the protein terminal domains. The alignment and consistency of the RMSF values across the triplicates, represented by the median line, confirm the reliability and reproducibility of the results. This consistency underscores the common area of flexibility or disorder in the AChE structure (Figure 11) and reinforces the stability of the analyzed complexes.

The analysis of the number of hydrogen bonds formed in each complex is presented in the graphs for AChE in complex with tacrine (Figure S5A, Supplementary Materials), compound **12** (Figure S5B, Supplementary Materials), and compound **13** (Figure S5C, Supplementary Materials). These results show the interactions and stability of these complexes over time.

In the AChE–tacrine complex, the number of hydrogen bonds fluctuates more significantly across the triplicates, with a median of approximately 1. This suggests fewer and less stable hydrogen bonds compared to involving the selenylated compounds. For the AChE–compound **12** complex, the number of hydrogen bonds shows moderate variation, with a median of around 2, indicating that compound **12** forms more stable hydrogen bonds with AChE compared to tacrine. Finally, the AChE–compound **13** complex demonstrates the highest number of hydrogen bonds, with a median of approximately 3. Additionally, the fluctuations are more moderate across the triplicates, suggesting that compound **13** forms the most stable hydrogen bonds with AChE among the tested ligands.

**Figure 12:**
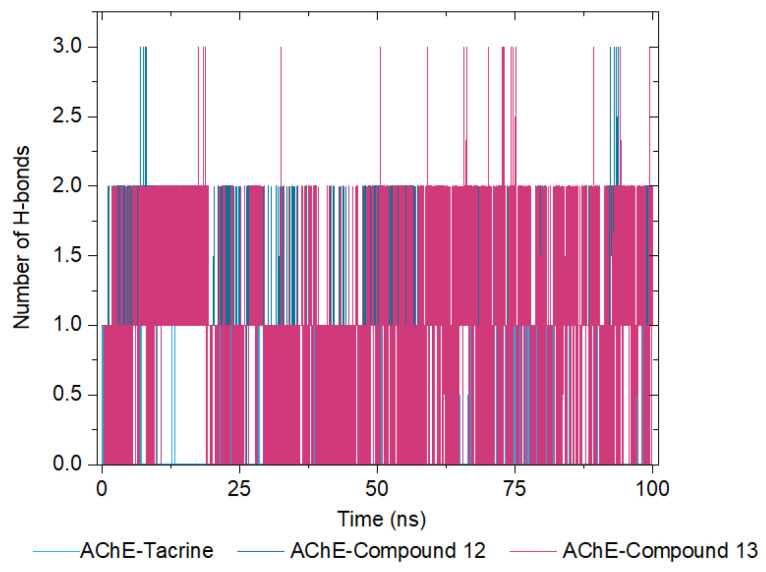
Number of Hydrogen Bonds Formed over Time for AChE in Complex with Tacrine (Soft Blue Line), Compound **12** (Dark Blue Line), and Compound **13** (Pink Line).

The medians across the triplicates for each complex are summarized in Figure 12, reinforcing the conclusion that AChE–compound **13** exhibits the most stable and numerous hydrogen bonds, followed by compound **12** and tacrine. These findings highlight the potential of compound **13** to form stable interactions with AChE, underscoring its efficacy as a promising ligand.

## 3. Materials and Methods

### 3.1. DFT Theoretical Studies and Calculations

Theoretical calculations were first performed on chemical structures in in silico studies. Determination of molecular structure, geometric optimization, and conformational studies were performed. The calculations were performed with the Semi-Empirical method PM3–Parameterized Model 3 by Stewart 1989 [39]. Computational programs employed in the analysis included the following: HyperChem 8.0-2002; ChemOffice-2005; GaussView 6.0 [36].

Computational techniques for DFT were employed in this work to ascertain the molecular geometries, focusing on the physicochemical properties and the most stable conformation with the lowest energy level, after which the molecular features of interest were examined [30]. The analysis of electronic behavior, reactivity, and stability characteristics was performed using B3LYP hybrid functional [40,42] at theoretical level 6-31++G (2d, 2p) [23]. These analytical processes were also performed in conjunction with GaussView and Gaussian 0.9 [35]. which facilitated proper modeling and thorough characterization of the molecules being studied.

### 3.2. Prediction of Pharmacokinetic Properties

The pharmacokinetic profiles of the designed derivatives were predicted using the PreADMET version 2.0 computational tool [24]. This web-based tool analyzes several pharmacokinetic parameters: human intestinal absorption (HIA), Caco-2 cell permeability Ca2 (Log*P* 8 × 10^−6^ ca^−2^ nm/s), blood–brain barrier penetration (BBBP), permeability into the central nervous system (CNS Log*PS*), plasma protein binding, P-glycoprotein interaction, and metabolism within both phases I and II [35]. These predictions give important insights into the process of absorption, distribution, and metabolic stability of the compounds [30].

### 3.3. Prediction of Toxicological Properties

The evaluation of toxicological profiles plays a crucial role in the early stages of drug design, ensuring that newly developed compounds exhibit minimal adverse effects. Computational tools such as PreADMET and other toxicology prediction software packages are commonly utilized to assess toxicological parameters of designed molecules. These tools predict potential toxicities, including hepatotoxicity, carcinogenicity, and mutagenicity, by analyzing structural features and comparing them to known toxic compounds [36].

By incorporating selenium into tacrine derivatives, it is anticipated that the therapeutic index will improve, as selenium is known for its antioxidative and detoxifying properties. Additionally, these predictions aim to identify any possible interactions with cytochrome P450 enzymes to further reduce risks associated with hepatotoxic metabolites, such as those seen with 7-hydroxytacrine. This approach ensures a safer pharmacological profile while maintaining efficacy [30].

### 3.4. Metabolic Predictions

Metabolic predictions are vital for understanding the biotransformation pathways of novel drug candidates and ensuring their stability and efficacy within biological systems. For tacrine derivatives, the primary focus is on Phase I and Phase II metabolic pathways, particularly involving cytochrome P450 enzymes. The incorporation of selenium into the tacrine scaffold may alter the metabolic stability and reduce the formation of toxic metabolites [35].

Computational tools, including PreADMET, facilitate the evaluation of critical parameters such as cytochrome P450 interaction, metabolic clearance rates, and the likelihood of forming reactive intermediates. These predictions are essential for assessing how the compounds will be absorbed, distributed, metabolized, and excreted, ultimately guiding further optimization of their pharmacokinetic profiles [23].

### 3.5. Interaction Analysis by Molecular Docking

Protein–ligand docking studies were carried out using the crystal structures of the target proteins Beta secretase 1 BACE1 (Homo sapiens, PDB: 4XSS) BACE2 (Homo sapiens, PDB: 1FKN), acetylcholinesterase AChE (Homo sapiens, PDB: 7E3I), and BuAChE (Homo sapiens, PDB 4BDS) extracted from the Protein Data Bank (PDB).

The molecular docking study was conducted with the aid of the AutoDock Vina version 1.1.2 program [26], and the PyRx version 0.8 graphical interface [27]. AutoDock is a set of tools designed to predict ligand–macromolecule interactions. To identify possible ligand–macromolecule binding configurations, the program offers 3 algorithm options: SA (Simulated Annealing), GA (Genetic Algorithm), and LGA (Lamarckian Genetic Algorithm). In this work, the LGA was selected as the search algorithm, as it has been shown to provide the best results in identifying the global minimum [28,30].

### 3.6. Molecular Dynamics Simulation

All simulations were performed using the all-atom molecular dynamics (MD) method with the Groningen Machine for Chemical Simulations (GROMACS) package, version 202 [29,31]. The initial poses of AChE–tacrine, AChE–compound **12**, and AChE–compound **13** were obtained from docking calculations. The simulations were conducted in a triclinic water box with periodic boundary conditions in all systems. The TIP3P water model [36] was employed to describe water molecules, and the net charge was neutralized by adding Na^+^ and Cl^−^ ions at a concentration of 0.15 M.

The AMBER99SB force field [32], included in the GROMACS package, was used for all system simulations. All systems were equilibrated in two stages: (i) using the canonical ensemble (NVT), with the constant number of particles, volume, and temperature, for 100 ps; and (ii) using the isothermal-isobaric set (NPT), with a constant number of particles, pressure, and temperature, for 1000 ps. The long-range electrostatic interactions were modeled for simulations with the Particle Mesh Ewald (PME) method [43].

Temperature coupling was achieved using a V-rescale thermostat [34] set at 310.15 K, while pressure control was maintained using a C-rescale barostat [44] during NPT equilibration and production, both with a reference pressure of 1 bar and compressibility of 4.5 × 10^−5^/bar. The LINCS algorithm constrained covalent bonds to their equilibrium length [41]. The integration steps of all simulations were set to 2 fs.

The ligand topology for each complex was prepared using the AnteChamber PYthon Parser version 3.0 interface (ACPYPE) server [45,46], which assigns charges and force field parameterizations to small organic molecules according to the Generalized Amber Force Field (GAFF). Ultimately, a 100 ns trajectory was obtained for each 3D complex. The root-mean-square deviation (RMSD) and root-mean-square fluctuation (RMSF) values were calculated to assess structural stability. The number of hydrogen bonds formed through the 100 ns also was analyzed.

## 4. Conclusions

In conclusion, this comprehensive investigation into tacrine and its selenium-modified derivatives has provided significant insights into their pharmacokinetic, toxicological, and molecular interaction profiles, paving the way for the development of safer and more effective treatments for neurodegenerative diseases like Alzheimer’s.

Furthermore, we noticed that the incorporation of selenium in these compounds notably modified the electronic shifts in the density of HOMO and LUMO; this suggests further, more in-depth studies should be performed to verify whether this characteristic is involved in probable toxicities of compounds containing selenium.

The theoretical studies, including Density Functional Theory (DFT) analyses, revealed that selenium-modified derivatives exhibit optimized electronic properties, such as narrower HOMO–LUMO energy gaps and balanced electronegativity. These modifications enhanced the reactivity and interaction potential of the compounds while maintaining sufficient stability to minimize undesirable side effects. Molecular docking studies further confirmed the enhanced binding affinity of the derivatives, particularly compounds **12** and **13**, to critical enzymes such as acetylcholinesterase (AChE), butyrylcholinesterase (BuAChE), and beta-secretases (BACE1 and BACE2). The interaction energies demonstrated that these derivatives form more stable complexes with their targets compared to tacrine, supporting their potential as effective inhibitors.

The pharmacokinetic evaluations indicated that the selenium-modified derivatives possess favorable absorption, distribution, metabolism, and excretion (ADME) profiles. Improved intestinal absorption and reduced interactions with plasma proteins enhance their bioavailability, while their ability to cross the blood–brain barrier makes them suitable for targeting central nervous system disorders. Moreover, the higher lipophilicity of the derivatives contributes to their enhanced membrane permeability, further increasing their therapeutic potential.

Toxicological predictions highlighted the significant advantages of selenium-modified derivatives over tacrine. The derivatives demonstrated reduced risks of hepatotoxicity and skin sensitization, which are major limitations of tacrine’s clinical use. While some derivatives showed plausible carcinogenicity, compounds like **8**, **12**, **13**, and **15** exhibited no toxicological concerns, making them particularly promising for further development.

Molecular dynamics simulations, including RMSD and RMSF analyses, provided additional evidence of the enhanced stability of the derivatives in complex with AChE. The selenium modifications were shown to stabilize key residues within the enzyme’s active site, reducing flexibility and ensuring strong binding interactions. Compound **13**, in particular, emerged as the most stable and effective derivative, demonstrating the significant benefits of rational molecular design.

Overall, the findings of this study underscore the potential of selenium-modified tacrine derivatives as next-generation therapeutics for Alzheimer’s disease. By addressing the limitations of traditional tacrine therapy, namely hepatotoxicity and instability, these derivatives offer a balanced approach that combines efficacy, safety, and stability. Future research should focus on experimental validation of these compounds, including in vitro and in vivo experiments, to confirm their therapeutic potential and advance their development into clinically viable treatments. The study also highlights the importance of integrating computational, theoretical, and experimental methodologies to optimize drug design and accelerate the discovery of innovative therapeutic agents.

## Figures and Tables

**Table 1:** Pharmacokinetic Properties of Tacrine and its Selenium-Modified Derivatives: HIA, BBBP, Permeability, and Log*P* Values.

Compounds	HIA (%)	BBBP (Log*CC*)	Permeability CNS (Log*PS*)	Permeability (Log*P* 8 × 10^−6^ ca^−2^ nm/s)	Log*P*
**1**	98.16	0.09	−1.42	1.54	3.99
**2**	98.60	0.06	−1.42	1.38	4.46
**3**	99.65	−0.09	−1.53	1.01	3.74
**4**	97.14	0.05	−1.42	1.39	4.51
**5**	98.15	0.04	−1.46	1.24	4.13
**6**	97.08	0.05	−1.42	0.99	4.78
**7**	97.30	0.06	−1.43	1.00	4.78
**8**	96.22	0.04	−1.40	1.01	4.87
**9**	99.94	−0.08	−1.54	1.02	3.74
**10**	98.88	0.08	−1.42	1.38	4.46
**11**	97.36	0.06	−1.42	0.99	3.78
**12**	98.63	0.02	−1.38	0.98	5.75
**13**	93.65	0.07	−1.36	1.01	4.99
**14**	98.97	−0.01	−1.23	0.97	5.39
**15**	100	−0.06	−1.13	0.99	4.15
**16**	95.24	−0.03	−1.73	1.47	3.51
**17**	98.13	0.12	−1.47	1.53	3.59
**18**	98.57	0.09	−1.46	1.38	4.06
**19**	98.12	0.07	−1.50	1.24	3.73
**Tacrine**	94.17	0.21	−1.70	1.58	2.83

For the HIA classification, 0~30% is considered poorly absorbed, 30~70% moderately absorbed, and 70~100% well absorbed. For high absorption in the central nervous system (CNS) Log*CC* above 0.3, for a medium absorption, 0.3~0.1, and for low absorption, less than −1. Compounds with Log*PS* greater than −2 have permeability into the CNS, while those with Log*PS* less than −3 do not penetrate the CNS. Log*P* 8 × 10^−6^ ca^−2^ nm/s), values 8 × 10^−6^ ca^−2^ nm/s are considered); values less than 0.1 indicate low permeability, values between 0.1~0.9, medium permeability, and values above 0.9, high permeability.

**Table 2:** Toxicological Predictions for Tacrine and Its Selenium-Modified Derivatives: Carcinogenicity, Hepatotoxicity, and Skin Sensitization.

Compounds	Carcinogenicity in Humans	Hepatotoxicity in Humans	Skin Sensitization
**1**	PLAUSIBLE	-	-
**2**	PLAUSIBLE	-	-
**3**	PLAUSIBLE	-	PLAUSIBLE
**4**	PLAUSIBLE	-	-
**5**	PLAUSIBLE	-	-
**6**	PLAUSIBLE	-	-
**7**	PLAUSIBLE	-	-
**8**	-	-	-
**9**	PLAUSIBLE	-	PLAUSIBLE
**10**	PLAUSIBLE	-	-
**11**	PLAUSIBLE	-	-
**12**	-	-	-
**13**	-	-	-
**14**	PLAUSIBLE	-	-
**15**	-	-	-
**16**	PLAUSIBLE	-	-
**17**	PLAUSIBLE	-	-
**18**	PLAUSIBLE	-	-
**19**	PLAUSIBLE	-	-
**Tacrine**	PLAUSIBLE	CERTAIN	PROBABLE

**Table 3:** Interaction Energies (ΔG) of Tacrine Derivatives with AChE, BuAChE, BACE1, and BACE2 Enzymes.

Compounds	∆G (−kcal/mol) (AChE)	∆G (−kcal/mol) (BuAChE)	∆G (−kcal/mol) (BACE1)	∆G (−kcal/mol) (BACE2)
**1**	−10.2	−9.8	−9.2	−9.0
**2**	−10.4	−10.1	−9.1	−8.9
**3**	−10.5	−9.3	−8.9	−8.9
**4**	−10.4	−9.5	−9.0	−8.8
**5**	−10.6	−9.6	−9.0	−8.9
**6**	−10.1	−9.6	−9.1	−8.8
**7**	−10.2	−9.8	−9.0	−8.9
**8**	−11.5	−10.7	−9.3	−9.6
**9**	−10.1	−9.5	−9.0	−8.9
**10**	−10.1	−9.8	−9.3	−9.2
**11**	−9.7	−10.1	−9.0	−8.8
**12**	−11.7	−11.9	−9.9	−9.7
**13**	−12.4	−12.9	−10.9	−9.9
**14**	−12.7	−10.8	−9.3	−9.5
**15**	−9.8	−11.2	−10.7	−9.1
**16**	−10.2	−8.0	−9.3	−7.5
**17**	−9.8	−9.6	−9.0	−8.7
**18**	−10.7	−9.9	−9.0	−8.7
**19**	−10.2	−9.7	−9.3	−9.1
**Tacrine**	−9.3	−8.2	−9.3	−7.7

## Data Availability

Data are contained within the article and Supplementary Materials.

## Supplementary Materials

The following supporting information can be downloaded at: https://www.mdpi.com/article/10.3390/molecules30122553/s1, Figure S1: HOMO and LUMO of compounds **1**–**18** are plotted below. The HOMO and LUMO distributions are highlighted in yellow and green color for the isosurface plots of electron density, respectively. Each molecule occupies one row, with HOMO on the left and LUMO on the right. Important functional groupswhich affect the distribution of molecular orbitals-were drawn to point out main structural and electronicdifferences among. where we can see that, in the compound where Seleniun is replaced by Oxygen, the total change in the distribution of HOMO shows that selenium might be mainly responsible for this distribution pattern. Figure S2: Validation of the molecular docking methodology of A AChE (Homo sapiens, PDB 7E3I) and B BACE1 (Homo sapiens, PDB 4XSS) with their respective crystallographic ligands (tacrine and WCA). Figure S3: RMSD triplicates of the AChE apoprotein (A) and its complexes with Tacrine (B, C), Compound **12** (D, E), and Compound **13** (F, G). The soft blue, dark blue, and pink lines represent runs 1, 2, and 3, respectively, while the red line indicates the median across the three runs. The RMSD of the AChE protein backbone (aligned to its own backbone) is shown in graphs A, B, D, and F. The RMSD of ligand heavy atoms (aligned to the protein backbone) is shown in graphs C, E, and G. Figure S4: RMSF triplicates of the backbone of AChE apoprotein (A) and its complexes with Tacrine (B), Compound **12** (C), and Compound **13** (D). The soft blue, dark blue, and pink lines represent runs 1, 2, and 3, respectively, while the red line indicates the median across the three runs. Figure S5: Number of hydrogen bonds triplicates formed over time for AChE in complex with Tacrine (A), Compound **12** (B), and Compound **13** (C). The soft blue, dark blue, and pink lines represent runs 1, 2, and 3, respectively, while the red line indicates the median across the three runs.
