# Supplementary material for: DFT-Based Elucidation and Evaluation of Selenium-Modified Tacrine Derivatives: Theoretical and Physicochemical Insights for Alzheimer’s Disease Therapy
Source: Molecules. 2025 Jun 11;30(12):2553. doi: 10.3390/molecules30122553 (PMC12196396; doi:10.3390/molecules30122553)
Supplement: Supplementary file 1 [file molecules-30-02553-s001.zip › molecules-3672195-supplementary.pdf]

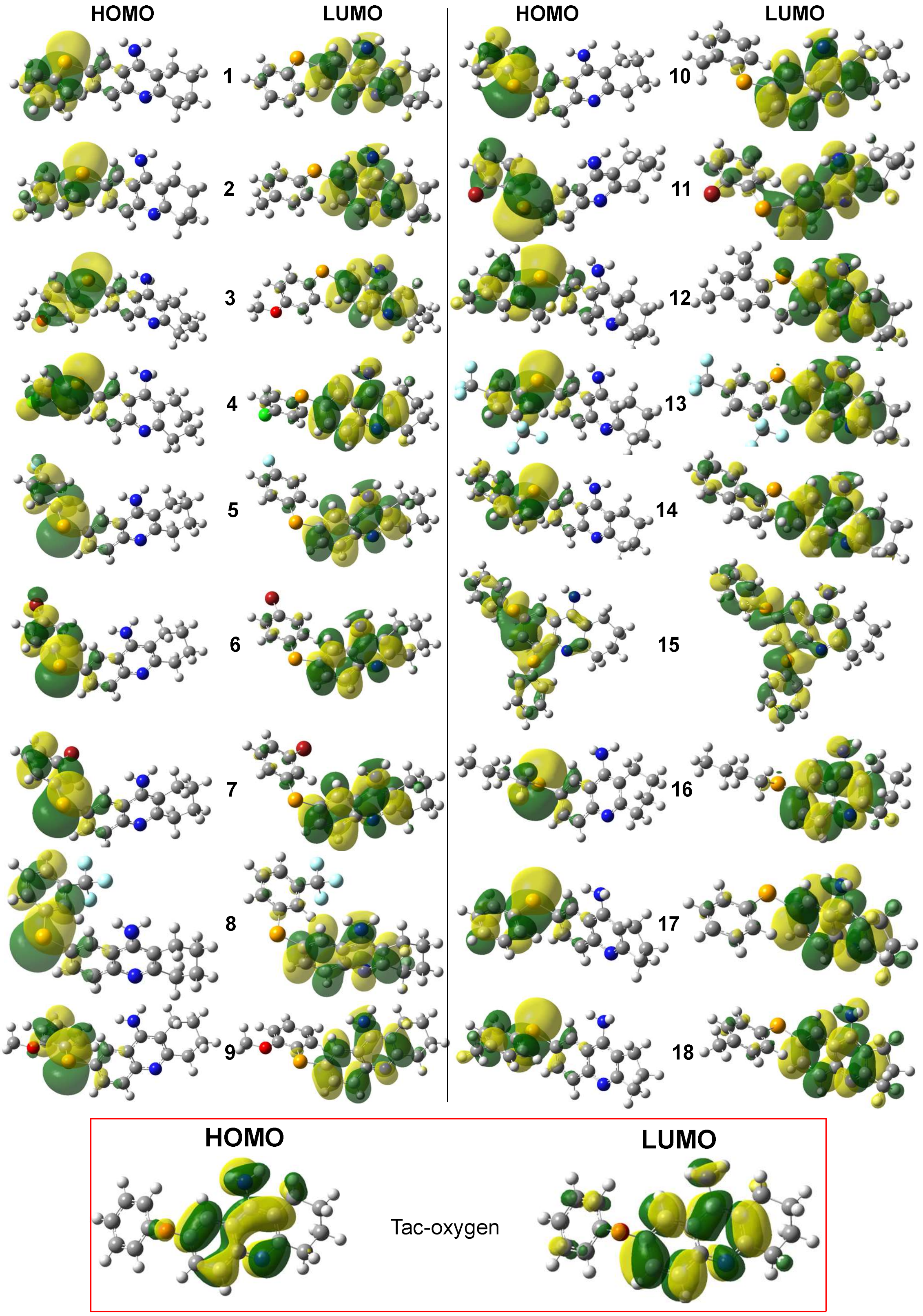

Figure S1: HOMO and LUMO of compounds **1-18** are plotted below. The HOMO and LUMO distributions are highlighted in yellow and green color for the isosurface plots of electron density, respectively. Each molecule occupies one row, with HOMO on the left and LUMO on the right. Important functional groups-which affect the distribution of molecular orbitals-were drawn to point out main structural and electronic differences among. where we can see that, in the compound where Seleniun is replaced by Oxygen, the total change in the distribution of HOMO shows that selenium might be mainly responsible for this distribution pattern.

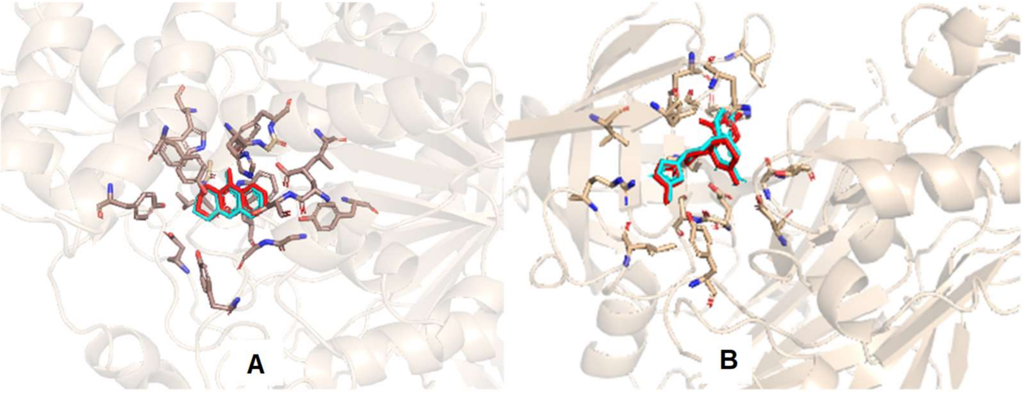

Figure S2: Validation of the molecular docking methodology of **A** AChE (Homo sapiens, **PDB** 7E3I) and **B** BACE1 (Homo sapiens, **PDB** 4XSS) with their respective crystallographic ligands (tacrine and WCA).

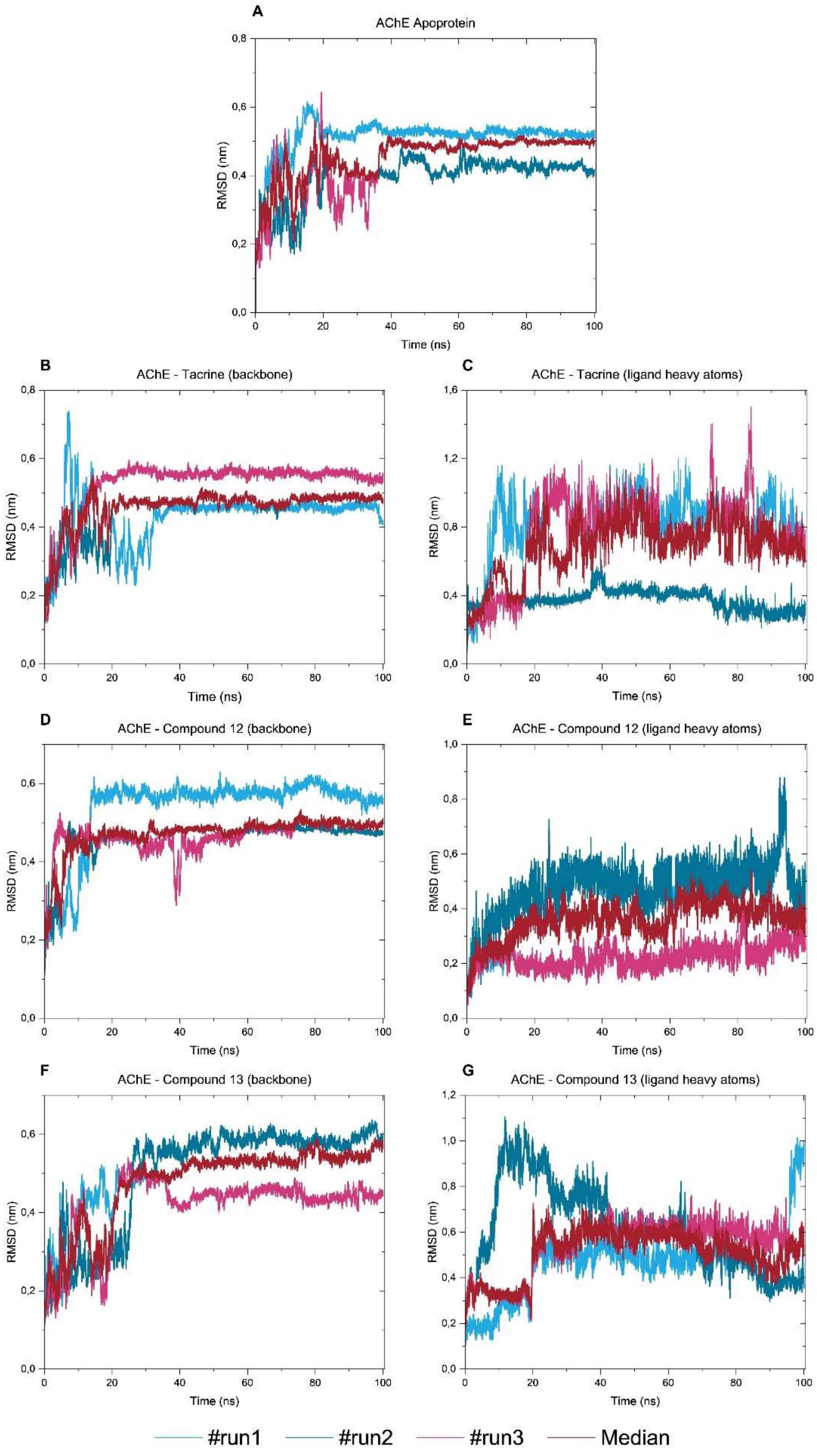

Figure S3: RMSD triplicates of the AChE apoprotein (A) and its complexes with Tacrine (B, C), Compound **12** (D, E), and Compound **13** (F, G). The soft blue, dark blue, and pink lines represent runs 1, 2, and 3, respectively, while the red line indicates the median across the three runs. The RMSD of the AChE protein backbone (aligned to its own backbone) is shown in graphs A, B, D, and F. The RMSD of ligand heavy atoms (aligned to the protein backbone) is shown in graphs C, E, and G.

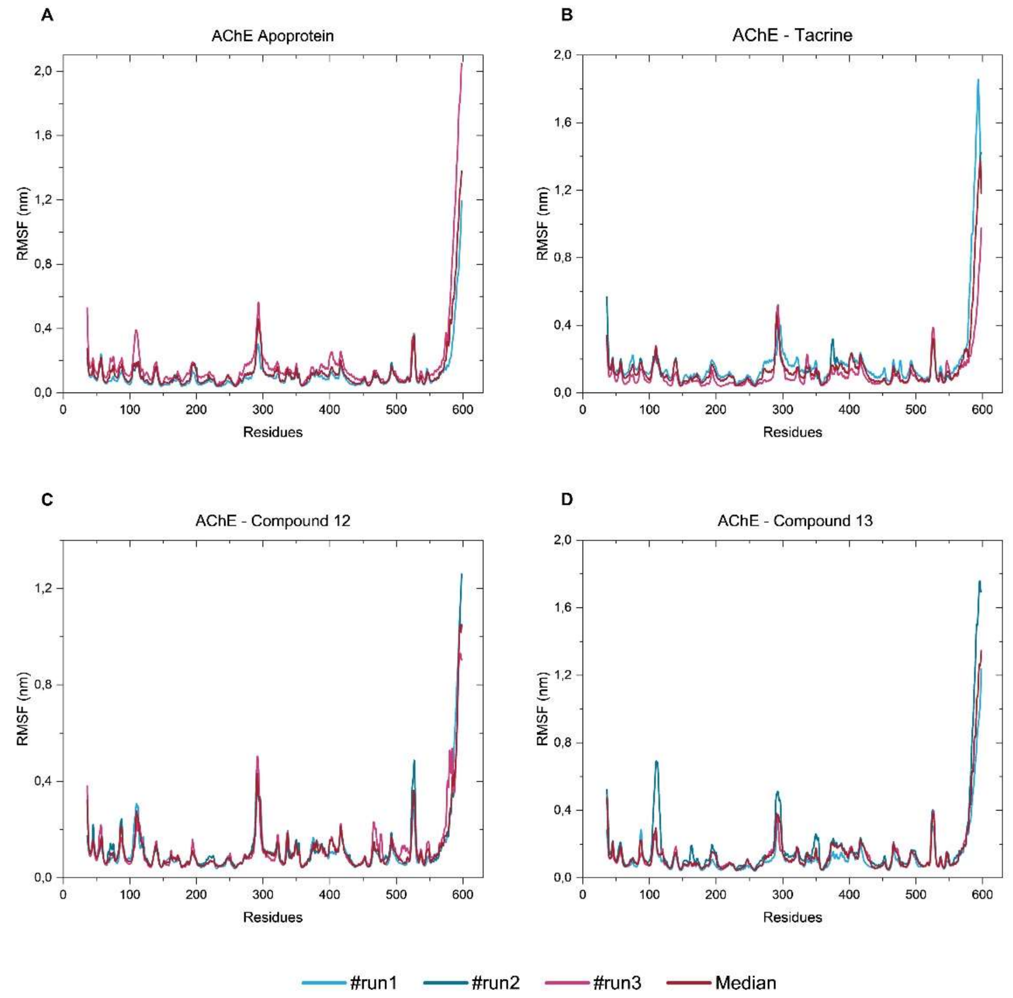

Figure S4: RMSF triplicates of the backbone of AChE apoprotein (A) and its complexes with Tacrine (B), Compound **12** (C), and Compound **13** (D). The soft blue, dark blue, and pink lines represent runs 1, 2, and 3, respectively, while the red line indicates the median across the three runs.

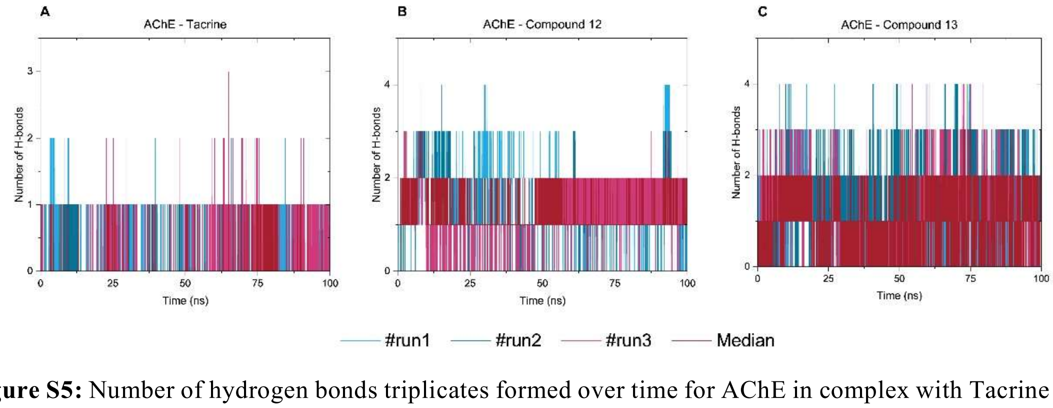

Figure S5: Number of hydrogen bonds triplicates formed over time for AChE in complex with Tacrine (A), Compound **12** (B), and Compound **13** (C). The soft blue, dark blue, and pink lines represent runs 1, 2, and 3, respectively, while the red line indicates the median across the three runs.
